# Riparian ecosystems in human cancers

**DOI:** 10.1111/eva.12015

**Published:** 2012-10-10

**Authors:** Khalid O Alfarouk, Muntaser E Ibrahim, Robert A Gatenby, Joel S Brown

**Affiliations:** 1 H. Lee Moffitt Cancer CenterTampa, FL, USA; 2 Institute of Endemic diseases, University of KhartoumKhartoum, Sudan; 3 Department of Biological Sciences, University of IllinoisChicago, IL, USA

**Keywords:** riparian zone, terrestrial-aquatic interactions, tumor biodiversity, tumor cords, tumor ecosystems

## Abstract

Intratumoral evolution produces extensive genetic heterogeneity in clinical cancers. This is generally attributed to an increased mutation rate that continually produces new genetically defined clonal lineages. Equally important are the interactions between the heritable traits of cancer cells and their microenvironment that produces natural selection favoring some clonal ‘species’ over others. That is, while mutations produce the heritable variation, environmental selection and cellular adaptation govern the strategies (and genotypes) that can proliferate within the tumor ecosystem. Here we ask: What are the dominant evolutionary forces in the cancer ecosystem? We propose that the tumor vascular network is a common and primary cause of intratumoral heterogeneity. Specifically, variations in blood flow result in variability in substrate, such as oxygen, and metabolites, such as acid, that serve as critical, but predictable, environmental selection forces. We examine the evolutionary and ecological consequences of variable blood flow by drawing an analogy to riparian habitats within desert landscapes. We propose that the phenotypic properties of cancer cells will exhibit predictable spatial variation within tumor phenotypes as a result of proximity to blood flow. Just as rivers in the desert create an abrupt shift from the lush, mesic riparian vegetation along the banks to sparser, xeric and dry-adapted plant species in the adjacent drylands, we expect blood vessels within tumors to promote similarly distinct communities of cancer cells that change abruptly with distance from the blood vessel. We propose vascular density and blood flow within a tumor as a primary evolutionary force governing variations in the phenotypic properties of cancer cells thus providing a unifying ecological framework for understanding intratumoral heterogeneity.

## Introduction

The clinical importance of evolution in producing significant intratumoral cellular heterogeneity is well recognized (Iwasa and Michor 2011; Gerlinger et al. 2012) but analysis of the primary evolutionary dynamics often focuses exclusively on genetic mutations (Temin 1984, 1988; O'Connell et al. 1994; Jackson and Loeb 1998; Hahn et al. 1999; Schl tterer 2000; Nowak et al. 2004, 2006; Iwasa et al. 2005; Michor et al. 2005; Durrett et al. 2010; Sottoriva et al. 2011). Here, we propose that a more complete application of Darwinian dynamics can substantially improve our understanding of intratumoral evolution. That is, while mutations produce the heritable variation, we note that environmental selection and cellular adaptation actually determine the strategies (and genotypes) found among cancer cells that persist and proliferate within their ecosystems (Alfarouk et al. 2011a). That is, intratumoral heterogeneity must reflect, in addition to genetic mutations, variations in interactions between cellular strategies (phenotypes) and environmental selection forces. We propose that the tumor vascular network is a common and primary cause of intratumoral heterogeneity. Specifically, variations in blood flow result in regional changes in substrates, such as oxygen (different of area, oxygenated, hypoxic, and anoxic), and metabolites, such as acid for example Lactate, that serve as critical microenvironmental selection forces (Gatenby and Gillies 2008; Alfarouk et al. 2011b). Here, we examine the evolutionary and ecological consequences of variable blood flow by drawing an analogy to riparian habitats within desert landscapes.

## Cancer and natural ecologies

In nature, ecosystems represent webs of interactions among and between biotic and abiotic components. Biotic components comprise the living systems that can evolve and co-evolve. These generally include plants, animals, fungi, protists, and bacteria. On the other hand, abiotic components include environmental factors such as rivers, soil, and temperature. These generally include the physical and climatic factors. Such ecosystems often exhibit substantial feedbacks between biotic and abiotic components as each influences the structure and dynamics of the other.

Similarly, clinical cancers can be viewed as ecosystems containing both evolving cancer cell populations and multiple host components including mesenchymal and immune cells, blood and lymphatic vessels, and extracellular matrices (Michelson et al. 1987; Gatenby 1991; Pienta et al. 2008). Cancer cells, within this conceptual framework, form the biotic components of the ecosystem, whereas abiotic components include blood flow, pH, temperature, intercellular fluids, and chemical fluxes (Huber et al. 2010). In this tissue ecosystem, we consider normal cells to be part of the abiotic environment. While normal cells are living entities of the whole organism, they mostly do not evolve or participate in an evolutionary game with the tumor cells. Within the ecological and evolutionary context of the tumor cells, normal cells are simply highly dynamic, changeable, and crucial elements of the cancer cell's habitat. So, while arguable, the mesenchymal, stromal, ductal, and/or epithelial cells within and around tumors can be viewed as highly interactive components of the cancer cells' ‘physical’ environment.

**Figure 1:**
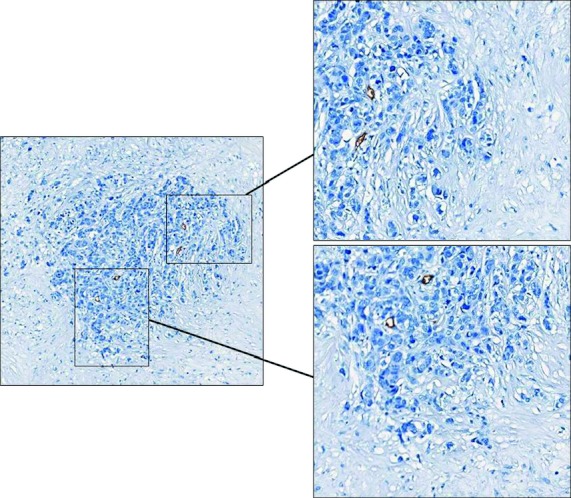
Micrograph of a invasive breast cancer following staining with hematoxylin and eosin and immunohistochemical stain for CD44 to identify endothelial cells forming blood vessel. In the two right panels, tumor cells are seen densely clustered around small blood vessels (stained brown). Distant to the blood vessels, there is an abrupt transition to regions of necrosis with sparse cellularity.

For cancer cells, a major role for the abiotic components of the tumor ecosystem is supply of nutrients and removal of metabolites (Vaupel et al. 1989). Previous work has clearly demonstrated the critical role of angiogenesis and blood flow in tumor growth and invasion (Zetter 1997; Folkman et al. #b^100^). In the absence of angiogenesis, tumors are limited in size to just a few millimeters in diameter. Thus, clinically relevant tumors cannot occur without the growth of blood vessels to deliver nutrients and remove metabolites (Pluda 1997). Thomlinson and Gray (1955) demonstrated the presence of ‘tumor cords’ in clinical cancers that represent cylinders of cellular growth around blood vessels (Fig. 1). Within this peri-vascular tissue, reaction–diffusion kinetics results in regions of hypoxia and acidosis (Gatenby and Gawlinski 1996). These regional variations in critical environmental factors produce distinct selection forces on the tumor cells inhabiting particular regions of a tumor. Tumor regions beyond about 100 µm from blood vessels experience very low concentrations of substrate and high concentrations of potentially toxic metabolites such as H^+^ (Gillies and Gatenby 2007). Such ‘physically’ harsh regions of the tumor are often described as ‘necrotic’ but in fact, they support sparse populations of cancer cells (Fig. 1) that are capable of repopulating the primary tumor and forming metastases (Bonfil et al. 1988).

Despite the significance of blood vessels for cancer cells, tumors typically possess rather chaotic blood flow from vessels that seem handicapped or malformed relative to normal vascularization (Runkel et al. 1995; Carmeliet and Jain 2000; Silva et al. 2009). As a result, highly variable relationships exist between angiogenesis, vascular tissue density, and blood flow. While aberrant relative to normal vasculature, intratumoral blood vessels are decisive for the proliferation, growth, and distribution of cancer cells within a tumor. Thus, distance from vascular tissue may be the first and primary determinant of microenvironmental heterogeneity within tumors.

## Riparian habitats in nature

Useful comparisons can generally be drawn between distinct ecosystems in nature. For instance, while quite different in origin and location, alpine flora and fauna (above the tree line of mountaintops) have striking similarities to the flora and fauna of tundras (a biome north of the boreal forests of North America, Scandanavia, and Russia). Similarly, we seek useful comparisons between tumor ecosystems and natural ones. In particular, we see an analogy between the arrangement of cancer cells around blood flow and vegetation around rivers within natural ecosystem. While other metaphors have been proposed for tumor dynamics (i.e., suburban sprawl around urban centers (Ryan et al. 2010)), we see direct, useful, and applicable parallels between tumor ecosystems and arid ecosystems with riparian zones.

**Figure 2:**
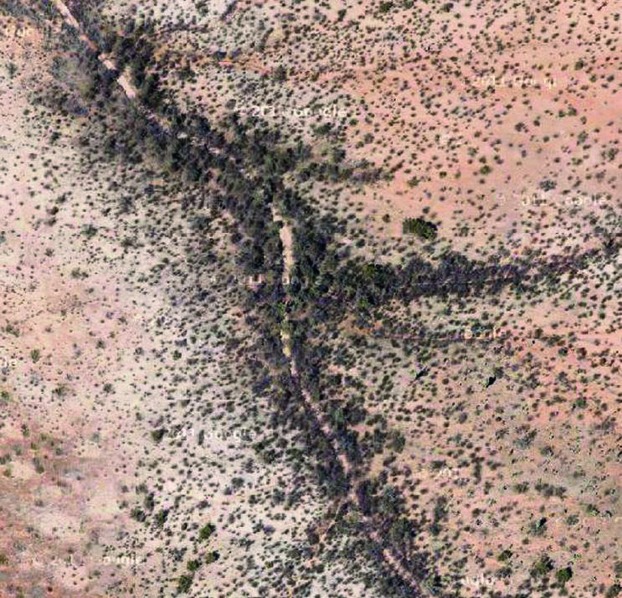
Riparian habitat in the Sonoran Desert of Arizona. The riparian zone is seen around the river beds and densely populated by mesquite and ironwood trees. There is an abrupt transition to the adjacent xeric zone, which is sparsely populated by creosote, bur-sage, and salt bushes (Satellite image from zoogle Maps).

Rivers, particularly those in desert or semi-arid landscapes, bring nutrients and resources to plants and carry away salts, toxic minerals, and plant metabolites. In an arid landscape, the edges of the river often support tall, lush vegetation including trees and tall shrubs. The vegetation region around a perennially or seasonally flowing river is known as the riparian zone or habitat (Fig. 2).

Desert riparian habitats produce zones of thick vegetation that occur along permanent or seasonal rivers flowing through otherwise dry landscapes (Gregory et al. 1991). The vegetation generally consists of thickets of shrubs and trees (trees include willows, mesquite, tamarisk, and cottonwoods), which differ in their height, and in the breadth and depth of their roots (Tufekcioglu et al. 1999). In desert riparian habitats, there is generally an abrupt transition from the ‘mesic’ shrubs and trees of the riparian zone and the ‘xeric’ grasses, shrubs, and cactus that occur in the drylands away from the stream (Harms et al. 2009). Away from the stream, the level and availability of groundwater decline and the accumulation of electrolytes such as calcium and sodium salts generally increases (Cui and Shao 2005; Yong-Zhong et al. 2012). Moreover, the riparian habitats themselves may be somewhat variable in the height of trees, the lushness of the vegetation, and the width of the riparian zone (Naiman and D′ecamps 1997a). This is because rivers, like blood vessels in a tumor, differ in velocity (fast- or slow-moving rivers), abiotic contents (e.g. minerals), water and temperature (Van der Velde et al. 2004). All of these factors shape the riparian zone. The riparian zone is characterized by complete and thick vegetation cover with multiple layers of vegetation (Kovalchik and Chitwood 1990; Naiman and Pollock 1993; Schade et al. 2002). It forms an environment that is abiotically benign in the sense of offering high nutrients, high moisture availability, and a low accumulation of potentially toxic salts – a phenomenon associated with ‘dilution zones’ (Naiman et al. 1997b; Gatti et al. 2006). Conversely, it is biotically harsh in the sense of intense competitive interactions between plants for space and nutrients (Casper and Jackson 1997; Naiman et al. 2000). The plants of the riparian zone, near the river, are strong competitors but are relatively intolerant of physical harshness (Glenn et al. 1998).

In deserts, the riparian region may be only 4–10 trees wide (perhaps 10–15 m wide). Beyond this point, there is an abrupt shift to much sparser vegetation that may only cover 10–30% of the aboveground space (Schade et al. 2002). The plants beyond the riparian zone are generally grasses and short shrubs (Naiman et al. 1997b) that experience a more biotically benign environment with less intense competition between plants for space and resources. That said the abiotic environment is significantly harsher having fewer nutrients, less water availability, and the accumulation of mineral salts up to toxic levels (Vaupel et al. 1989). In just a short distance from the river, the vegetation changes from the mesic species of the riparian zone to a sparsely populated community of dry or xeric species. The mesic species of the riparian zone are different and distinct from the xeric species of the adjacent drylands. There is little to no overlap between these species.

The aridity, lack of nutrients, and salt buildups preclude the riparian species from moving into and inhabiting the drylands between streams. The competitive superiority of the riparian plant species prevents the xeric species from invading the narrow riparian zone along the river banks. As one moves still farther from rivers, or into areas where water runoff simply evaporates, the soil may become so impregnated with sodium, calcium, and other salts that no plants can grow at all – these constitute the sterile, ‘necrotic’ salt pans of desert ecosystems.

## Riparian habitats in cancer

The striking similarity between the arrangement of cancer cells around blood vessels and vegetation around waterways leads us to consider tumor ecosystems within the context of the biotic and abiotic interactions in riparian ecosystems. The adaptations and ecologies of riparian zones in arid ecosystems can provide insights into the ecological and evolutionary dynamics of tumor cords. Specifically, we suggest that tumor cords, like desert riparian habitats, are governed by counter gradients of abiotic and biotic rigor. These ‘predictable’ regions offer opportunities and hazards to cancer cells and promote the evolution of region-specific cancer cell phenotypes and their corresponding genotypes (Cuny et al. 2000; Wakimoto et al. 2012).

### Perivascular tumor – abiotically benign, biotically harsh

**Figure 3:**
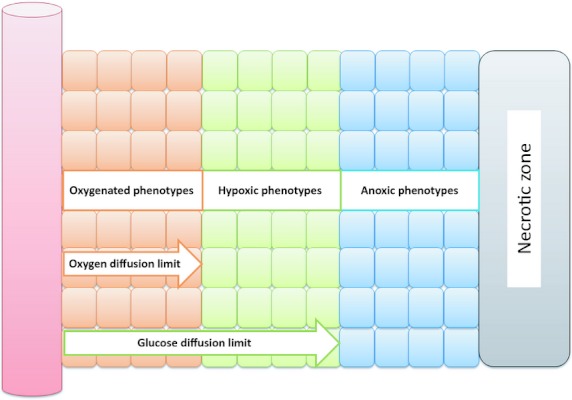
Schematic of the abiotic gradients around intratumoral blood vessels that select for specific phenotypic adaptations.

Helmlinger et al. (1997) measured the spatial heterogeneity of pO_2_ and pH around tumor blood vessels (summarized in Fig. 3). These are critical elements of the abiotic perivascular ecology. The measurements, consistent with the prior theoretical analysis by Krogh and Tomlinson and Gray, demonstrated a gradually decreasing pO_2_ and pH with distance from blood vessels (Helmlinger et al. 1997). By examining the correlation between pO_2_ and pH changes, they inferred a gradient of glucose concentration as well. Typically, the tumor microenvironment becomes too harsh (due to low pO_2_ and pH) to support dense cellular populations within about 150 µm of the blood vessels (Fig. 1). Like the trees of desert riparian ecosystems, this results in a riparian zone of tumor cells that is about 4–8 cells wide. Beyond this zone, tumor cells experience considerable abiotic rigor akin to the drylands just beyond the trees of desert riparian habitats.

These predictable, consistent heterogeneous distributions of oxygen, pH, and glucose clearly produce regional variations in the tumor microenvironment. As shown in Fig. 1, tumor cells cluster around vessels for the same reasons that vegetation clusters around waterways in riparian zones. What are the expected ecological and evolutionary consequences for cancer cells growing in abiotically benign but biotically rigorous regions? We propose that the analogy has three important consequences for tumor biology:

1. The riparian zone in tumors (as in nature) will be densely populated by cancer cells that are highly adapted to abiotically ‘rich’ environments. Typically, cancer cells that grow in these mesic regions will be highly competitive, characterized by high substrate uptake and utilization, rapid proliferation, and less resistant to apoptosis (Arends et al. 1994). However, they will be extremely intolerant of and susceptible to declines in resource concentrations. Thus, we expect the cancer cells of the riparian zone to form the densest and most abundant populations. Hence, these mesic ‘species’ of cancer cells will be the ones most often observed, and they will be disproportionately represented in, for example, random tumor biopsies.
2. Riparian zone tumor cells will be highly adapted to their circumstances but also less flexible and less phenotypically plastic (Hendrix et al. 2003). As a result, they will be less able to acclimate to new hazards or sudden losses of resources and substrate. The tumor cells of the riparian zone will, thus, be most vulnerable to an environmental perturbation such as chemotherapy or antiangiogenesis. Such riparian species lose twice from either the poisoning or drying of the ‘river’. They are the ones that are most exposed due to their proximity to the blood vessels, and these cells are the least able to acclimate to the changed circumstances.
3. Riparian zones from different continents or deserts generally have separate species of trees and shrubs that can sometimes be phylogenetically unrelated. Yet, the plants from these different systems exhibit similar evolutionary strategies and converge on similar ecologies. That is, all populations within the mesic regions demonstrate near identical properties in that they are highly adapted to a rich resource environment, proliferate rapidly, and form dense populations. Although riparian zone vegetation exhibit similar evolutionary strategies, they are typically different species! That is, at a genetic level, riparian populations are highly variable. However, they will exhibit the same ecological and phenotypic properties. Hence, when comparing riparian tumor cells from different tumors or from different patients, we should not be surprised to see considerable genetic variation. Yet, when making such comparisons, we predict striking concordances in the adaptations of these cancer cells for their respective riparian zones. In other words, while extensive genetic heterogeneity is found in cancer cells within and between tumors, analysis at the strategy or phenotypic level should actually find a convergence of cancer cells based on their distance from blood vessels. This will not be evident in the molecular analysis because the cellular strategies and phenotypes will supervene their genotypes (Gatenby et al. 2011). That is, different genotypes can result in identical adaptations to environmental selection forces.

### Xeric tumor populations – distant from blood vessel with harsh abiotic conditions

In desert landscapes, regions adjacent to riparian zones typically exhibit harsh environmental conditions. The plant populations invariably maintain very low population densities with phenotypes that are highly efficient at using sparse resources and phenotypes (e.g., saltbush) that can tolerate environmental conditions that are lethal to mesic species. In the equivalent xeric regions of a tumor, we expect the following:

1. Xeric populations of cancer cells should be different and distinct species from those in the riparian zones. While adjacent mesic and xeric habitats might favor a single generalist species capable of living in both habitats (Brown 1996), this is rarely seen in desert riparian systems. Rather, distinct species assemblages occupy each region. Species from one region cannot invade the other. Because the tumor population distant from blood vessels may be fairly sparse, these distinct populations may be undetectably small in biopsy specimens. Like xeric plants of the desert, we expect these xeric tumor cells to be hardy, flexible, phenotypically plastic and able to acclimate to shifts in harshness. The xeric tumor cells may be unusually resistant to apoptosis. We propose that these cells play a large and disproportionate role in tumor regrowth and responses to therapy.
2. Xeric species are less vulnerable to microenvironmental perturbations such as those from systemic cancer therapy. First, because they are far from the blood vessels, these tumor cells experience lower concentrations of any administered drug. The necessarily lower profusion of chemotherapy into the xeric region may facilitate the evolution of treatment resistance by the xeric cells. Second, the higher pH of the xeric regions may reduce the effectiveness and activity of weakly basic drugs such as doxorubicin (Raghunand and Gillies 2000; Mahoney et al. 2003; Trédan et al. 2007). Third, xeric species typically proliferate very slowly – rendering them relatively invulnerable to cell cycle–specific drugs. Hence, therapies may ‘lose’ in three ways against these cells.
3. Agents that alter tumor vasculature (Siemann 2006) may actually favor the tumor cells adapted to the xeric zones by reducing or eliminating riparian zones. And, finally, because the riparian tumor cells competitively exclude the xeric tumor cells from prime habitats, the disproportionate decline of riparian cells from treatment may indirectly benefit the xeric-adapted cancer cells.

## Conclusions

Over three decades ago, Nowell (1976) introduced an evolutionary paradigm for understanding tumor development and growth. Since, it is increasingly apparent that Darwinian dynamics lead to significant intratumoral heterogeneity (Merlo et al. 2006; Gerlinger et al. 2012). However, cancer biology often views evolution through an exclusively genetic context – emphasizing the role of accumulating mutations as the driving force of cancer cell proliferation and heterogeneity. We point out that, while mutations produce the heritable variation necessary for evolution, environmental selection and adaptations by tumor cells actually govern the strategies (and genotypes) that proliferate and form the cellular populations of a cancer. Thus, while molecular analysis generates an almost hopelessly complex heterogeneity in tumor cell genotypes, we propose that ultimately tumor phenotypes and genotypes must represent a set of predictable adaptations to a limited number of intratumoral environmental conditions. Thus, an appreciation for the ecological and evolutionary dynamics that occur within ecosystems (natural ones as well as in tumors) provides a unifying paradigm. That is, we propose that examining cancers as ecosystems allows a greater appreciation for the similarities among tumors, and the environmental selection forces that maintain genetic and phenotypic variability.

This led us to examine possible insights into tumor biology that can be gained from comparing natural ecosystems to tumor ecosystems. Of the manifold diversity of ecosystems found in nature, we suggest that desert riparian ecosystems share much in common with tumors. We propose that tumor cords recapitulate the biotic and abiotic gradients found in the mesic and xeric environments created by rivers in deserts. We predict that the evolutionary strategies of tumor cells should closely mimic those found in and around riparian habitats. Perhaps most importantly, we suggest that these dynamics represent a common evolutionary pathway within tumors. That is, while tumor cells within and between tumors typically show striking genetic diversity, we propose that convergent phenotypes will be found in the perivascular (mesic) and necrotic (xeric) regions of tumors. An understanding of tumor riparian ecosystems provides a framework for understanding key elements of tumor evolution and tumor responses to therapy.
